# Policy questions as a guide for health systems’ performance comparisons

**DOI:** 10.2471/BLT.24.291635

**Published:** 2024-06-04

**Authors:** Irene Papanicolas, Jonathan Cylus, Hugh Alderwick, Luca Lorenzoni

**Affiliations:** a Department of Health Services, Policy and Practice, Brown University School of Public Health, Providence, United States of America.; b European Observatory on Health Systems and Policies, London, England.; c The Health Foundation, London, England.; d Organisation for Economic Co-operation and Development, 2 Rue André-Pascal, 75775 Paris CEDEX 16, France.

Researchers and policy-makers have long compared health system performance.^1^^,^^2^ International comparisons raise awareness of health systems’ relative strengths and shortcomings, prompting policy debates and informing policy decisions. Yet determining how these international comparisons can be used to improve health system performance is challenging. Health systems can differ in many ways, including how they are governed, how they are funded, how they generate and deploy resources, and how they deliver services.^3^^,^^4^ While the international health community widely agrees that these functions influence health system performance,^5^ understanding of how much they matter, which ones matter most and how they are affected by the context in which they operate remains limited. To gain relevant and meaningful insights from health systems comparisons that offer lessons for policy, we must agree on how to compare health systems. In this article, we argue that doing so requires collecting better, more granular data on a broad range of health system characteristics and using those data to choose the most appropriate health system comparators.

## How are health systems compared?

Comparisons are often made between health systems that are deemed to share similar characteristics such as geographical location, historical legacy, income level, type of financing and public sector values. The desire to compare similar systems could stem from the idea that if health systems differ in too many ways, attributing any one characteristic to variations in performance would be challenging. Moreover, borrowing policy options from a health system that shares few common traits with that of its intended recipient is unlikely to lead to similar outcomes and may have unintended consequences. However, finding new solutions to complex problems is difficult if we only compare systems that approach their challenges in similar ways, while comparing health systems that differ markedly from one another in their design may bring important insights. Such comparisons among health systems with different design features could help policy-makers gauge if certain institutional features are consistently associated with better outcomes, and help to develop a broader evidence base for reform.^6^^,^^7^

Whether the aim is to compare similar systems or to compare health systems with different design features to each other, we need conceptual clarity on how to categorize or classify health systems to inform the selection of relevant comparators. Despite recognition of the multiple ways in which health systems differ, only a few broad categorizations used in comparative research exist. Too often, international health systems are categorized based on an oversimplified typology, either as social health insurance or as tax-financed system. These types are known as Bismarck and Beveridge, respectively (Otto von Bismarck instituted the first social health insurance model in Germany in 1883. William Beveridge established the national health service in the United Kingdom of Great Britain and Northern Ireland in 1948). While this typology may have been relevant historically to describe different ideologies for health system designs in the first half of the 20th century, it has become less meaningful over time. For example, to expand population coverage, many traditional social health insurance systems now use funds generated from general taxation to cover uninsured groups (such as the unemployed) who are unable to pay social insurance contributions. This results in social health insurance systems becoming increasingly dependent on general tax financing, blurring the distinction between Beveridge and Bismarck typology. As an illustration, while the Health Insurance Institute of Slovenia receives more than 95% of its annual funding from social insurance contributions, Hungary’s National Health Insurance Fund is largely financed by general taxation. Moreover, as health system comparisons increasingly include countries that do not share inherited features, this taxonomy has proven difficult to apply.

This point is further demonstrated in Table 1. Systems that are often described as Bismarck or Beveridge differ with regards to several other characteristics such as the way they pay providers, whether they have gatekeeping, how they incentivize quality and whether they use health technology assessments.

**Table 1:** Sample of health system characteristics for selected OECD countries

Country	Typology	Predominant form of hospital payment	Direct access to specialist care	Financial incentives to providers to enhance quality of care	Use of health technology assessment to inform coverage of medicines
France	Social health insurance (Bismarck)	Diagnosis-related group	Limited	Yes	Systematically used
Germany	Social health insurance (Bismarck)	Diagnosis-related group	Yes	No	Systematically used
Italy	Tax-financed system (Beveridge)	Diagnosis-related group	Limited	No	Used in some circumstances
Kingdom of the Netherlands	Social health insurance (Bismarck)	Diagnosis-related group	Limited	Some	Systematically used
Republic of Korea	Social health insurance (Bismarck)	Fee-for-service	Limited	Yes	Systematically used
Spain	Tax-financed system (Beveridge)	Global budget	Limited	Yes	Systematically used
Sweden	Tax-financed system (Beveridge)	Global budget	Yes	Yes	Systematically used
United Kingdom	Tax-financed system (Beveridge)	Budget and diagnosis-related group	No	Yes	Used in some circumstances

OECD: Organisation for Economic Co-operation and Development.

Source: OECD Health Statistics, 2024.^8^

## Choice of comparators 

Health systems are currently facing many challenges such as ageing populations and increasing multimorbidity; finding new ways to pay for expensive breakthrough treatments; and the increasing frequency of global threats to population health, such as pandemics and climate change. Governments approach these problems in different ways. For example, to incentivize better care for an older and unhealthier population, numerous governments are experimenting with innovating payment and delivery models in primary and secondary care. To address concerns about the costs and utilization of new technologies, many governments have introduced regulatory bodies that review and assess the effectiveness and sometimes cost-effectiveness of new technologies. During the coronavirus disease 2019 (COVID-19) pandemic, governments took varied approaches to prevent the spread of the virus, protect the health system from surges of demand, and incentivize rapid deployment of vaccines and treatments.

Do different approaches to common problems have differential effects on health system performance? And do some countries implement specific policies more effectively than others? Cross-country comparisons can help to answer these questions. However, to make the best use of comparative analysis, researchers need to select the adequate comparator countries. Policy-makers also have a range of priorities that may require different comparators depending on the question being asked. Choosing the most appropriate comparator countries for a particular policy question requires data on all essential functions of health systems. That is, distinguishing between systems based on whether they are Beveridge or Bismarck is not appropriate to inform all comparative health policy questions.

## Conceptual tools and data 

Any attempts to group health systems are only as good as the available data, agreed-on definitions underpinning these data, and approach taken to clustering. Groupings that are too broad risk masking complexity and variation between systems, while too much detail on variation in characteristics may result in health systems appearing to have no peers, reducing the scope for cross-country learning. The challenge lies in finding the optimal balance to ensure comparisons are meaningful, valid and policy relevant.

Much development in the conceptual and data tools to construct health system typologies has occurred in the past few decades. Information on health system inputs, such as expenditures by type and function or numbers of beds and doctors, has become increasingly harmonized and readily available through large intergovernmental organizations including the World Health Organization (WHO), the World Bank and the Organisation for Economic Cooperation and Development (OECD). This information has also begun to be supplemented with additional qualitative and survey data that consider how these inputs are used to meet health systems objectives, through initiatives such as the European Observatory’s Health System in Transition profiles, the OECD’s Health System Characteristics Survey and the WHO Health Financing Progress Matrix.^9^^–^^11^ These tools can help us group countries based on questions such as how health-care providers are recruited and trained, what processes are in place for ensuring minimum quality standards and what care similar patients are entitled to across countries, among others.

The demand for more evidence on the effects of public health policy on outcomes during the COVID-19 pandemic further catalysed data collection in this area. Multiple data sets were created to capture information on policies adopted by countries to combat the spread of the virus, such as the Blavatnik School of Government’s Oxford COVID-19 Government Response Tracker,^12^ a tool numerously cited in studies comparing the effectiveness of different national COVID-19 response tools. This example alone serves to highlight the vast demand for new information that can be used to classify health systems and compare policy responses across countries. As information capturing differences in how health systems work becomes more widely available, more must be done to better understand the variability in these characteristics and their association with performance.

## Conclusion

Health systems across the globe face major common challenges including demographic pressures, funding constraints, workforce gaps and rising inequalities. International comparisons can and should help policy-makers by providing much-needed information on which policies and design features can be most effective at addressing these challenges. But too often comparative analyses fail to provide meaningful information to inform policy; sometimes they can even confuse or mislead. We believe part of the problem lies in how we categorize health systems. When simple constructs are used to group countries, we risk obscuring the mechanisms and design features that are most relevant and responsible for variations in performance. Hence comparisons are needed that cluster and compare specific, policy-modifiable aspects of health systems – such as their governance, financing, the generation and deployment of resources, and the design of care delivery – aspects that are identified based on the policy question. More targeted classifications can further research, practice and policy by using more granular, internationally comparable available data that allow for asking better questions, learning from one another and informing reform options.
